# Implementation of ATP and Microbial Indicator Testing for Hygiene Monitoring in a Tofu Production Facility Improves Product Quality and Hygienic Conditions of Food Contact Surfaces: a Case Study

**DOI:** 10.1128/AEM.02278-20

**Published:** 2021-02-12

**Authors:** Jonathan H. Sogin, Gabriela Lopez-Velasco, Burcu Yordem, Cari K. Lingle, John M. David, Mario Çobo, Randy W. Worobo

**Affiliations:** a Department of Food Science, Cornell University, Ithaca, New York, USA; b 3M Company, St. Paul, Minnesota, USA; University of Helsinki

**Keywords:** hygiene monitoring, food quality, ATP luminometer, rapid, food production

## Abstract

Rapid ATP testing and microbiological enumeration are two common methods to monitor the effectiveness of cleaning and sanitation in the food industry. In this study, ATP testing and microbiological enumeration were implemented at a tofu production facility with the goal of improving cleaning practices and overall plant hygiene. Results from ATP monitoring were used to target areas of the production environment needing additional cleaning; ATP results were verified by microbiological enumeration of aerobic microorganisms, lactic acid bacteria, and yeasts and molds. Products from the production line were enumerated for the same microorganisms to determine if there was an impact on product quality. After the implementation of ATP monitoring and targeted cleaning, there was a statistically lower proportion of swabs that failed to meet established sanitary requirements for ATP, aerobic microorganisms, and lactic acid bacteria (*P* < 0.05) but not for yeasts and molds. ATP swabs and microbiological enumeration agreed on site hygiene 75.1% (95% confidence interval [CI], 72.3% to 77.7%) of the time. Product data indicated that unpasteurized finished products contained a statistically lower microbial load of the three groups of organisms following implementation of the practices (*P* < 0.05).

**IMPORTANCE** Cleaning and sanitation are critical to maintaining safe and high-quality food production. Monitoring these activities is important to ensure proper execution of procedure and to assure compliance with regulatory guidelines. The results from monitoring activities can direct targeted cleaning of areas with higher risk of contamination from foodstuffs and microorganisms. The results of this study show that ATP monitoring and microbiological enumeration are useful tools to verify and improve the efficacy of cleaning and sanitation practices, which can have a positive impact on both plant hygiene and product quality. However, testing regimes and critical parameters will vary based on the product and facility.

## Abstract (precis)

Cleaning and sanitation are critical to maintaining safe and high-quality food production. Monitoring these activities is important to ensure proper execution of procedure and to assure compliance with regulatory guidelines.

## INTRODUCTION

Many factors contribute to the microbial ecology of a food processing plant, including the product, processing steps, and scale of the operation. However, all food processing facilities regardless of product or size must maintain a sanitary processing environment (9 CFR 416; 21 CFR 117) (1, 2). Companies execute sanitary standard operating procedures (SSOPs) to reduce and control for the infiltration, movement, and growth of microorganisms in a plant (e.g., use of personal protective equipment, water treatment, air filtering, pest exclusion), but these programs do not and cannot completely prevent microorganisms from entering the processing environment. A major component of SSOPs involves cleaning and sanitizing the processing line and surrounding areas. Both the Food and Drug Administration and United States Department of Agriculture (USDA) require that food contact and non-food contact surfaces be cleaned and sanitized as frequently as necessary to prevent contamination of products (9 CFR 416.4; 21 CFR 117.35) (3, 4). These rules exist to prevent product contamination with hazards, i.e., “any biological, chemical (including radiological), or physical agent that has the potential to cause illness or injury” (21 CFR 117.3; see 9 CFR 417.1 for equivalent USDA definition) (5, 6). However, cleaning and sanitation (as well as other SSOPs) also control for spoilage microorganisms.

Cleaning and sanitation involve removal of material buildup from surfaces and subsequent application of a substance to reduce target microorganisms to an acceptable level. Cleaning should specifically remove proteins, carbohydrates, FOG (fats, oils, and grease), minerals, and water (7). These substances are growth substrates for various pathogenic and nonpathogenic microorganisms and quench the effectiveness of sanitizers by serving as off-target substrates for sanitizing compounds. Thus, sanitation is only effective if adequate cleaning precedes it. Cleaning and sanitation occur before and after production runs by a qualified and trained team; efforts are prioritized based on production zones, distinguished by likelihood to contaminate food. Zone 1 corresponds to food contact surfaces (highest risk of introducing contamination), zone 2 corresponds to non-food contact surfaces near zone 1, zone 3 corresponds to more distant non-food contact surfaces than zone 2, and zone 4 corresponds to surfaces outside of the production room (lowest risk of introducing contamination) (8). An individual on the cleaning and sanitation team utilizes visual inspection and timers to monitor progress and determine the endpoint of cleaning and sanitation activities. However, visual inspection has limited effectiveness, as food residues and/or microorganisms may be present even on a visually clean surface (9). Visual inspection should be an expectation, but companies use other verification methodologies to verify the efficacy of cleaning and sanitation.

Hygiene monitoring is the regular, systematic, and site-specific testing of a processing plant for an attribute relevant to the processing environment to verify the efficacy of a sanitation program or overall cleanliness of the plant (10). Hygiene monitoring differs from environmental monitoring in that it does not identify specific organisms, namely, environmental pathogens, but rather detects broad groups of microorganisms and/or food residues relevant to the processing environment, thus enabling the identification of sites that can harbor or support the growth of microorganisms due to the presence of food residues. Despite its nonspecific nature, hygiene monitoring is an important activity that supports the effectiveness of both food safety and food quality programs. Two common hygiene monitoring methods are culture-based quantification assays and rapid indicator testing for biological markers.

Culture-based enumeration provides quantification of microorganisms at a site but is biased due to sampling procedure, processing, and growth conditions (e.g., media composition, growth temperature, growth time) (11). The use of different growth media for hygiene monitoring selects for various groups of microorganisms but not individual taxonomic clades. Unlike environmental monitoring, hygiene monitoring seldom integrates sequencing tools (i.e., Sanger sequencing and next-generation platforms) for regular use; nonetheless, and beneficially, culture-based enumeration allows for the isolation and identification of suspected spoilage and/or residential microorganisms. Because culture-based methods rely on the growth of microorganisms, results are obtained after a minimum of 24 to 72 h, depending on the group of microorganisms in question (12, 13). Thus, enumeration-based monitoring tools lead to strictly reactive, but not real-time, solutions to deviations from cleaning and sanitation, delaying implementation of corrective actions when improper cleaning has been performed. Further, enumeration requires use of a dedicated laboratory space, trained personnel to process samples, and the purchase of several additional laboratory materials (e.g., growth media, incubators, pipettes), so only companies able to regularly pay for and manage or outsource these activities can effectively utilize culture-based monitoring tools.

Conversely, the use of rapid tests can be used to detect for the presence of biological markers resulting from microbial metabolism or food. Rapid tests are easy to conduct, fast, portable, and can direct real-time improvements to cleaning and sanitation regimes. Unlike culture-based monitoring assays, rapid tools do not require a dedicated laboratory space nor extensively trained personnel to conduct the tests. One of the most common rapid methodologies quantifies ATP, which is a molecule produced by all living cells and detected via an enzymatic assay utilizing a luciferin/luciferase complex to produce light (14). Light production can be measured as relative light units (RLU), thus converting ATP levels at individual sites to numerical values. The amount of light produced is directly proportional to the level of ATP and therefore used to assess the cleanliness of a site. Compared to culture-based methods, ATP detection has no sensitivity for specific groups of microbes (spoilage and/or pathogenic) because the structure of ATP is identical in all cells; ATP in food processing environments is derived from microorganisms, food residues, and other organic matter (15). Therefore, it can help identify niche sites where food is not efficiently removed during cleaning and sanitation (15). ATP detection is a more rapid (providing results in minutes) and accessible tool to a greater range of food production facilities for cleaning verification and hygiene monitoring compared to culture-based enumeration. However, ATP monitoring, like culture-based methods, is susceptible to sampling biases (e.g., swabbing pattern, swabbing area, surface characteristics), degradation of ATP or interference with the assay due to cleaning and sanitation compounds, and inability to effectively detect spores (16).

**FIG 1:**
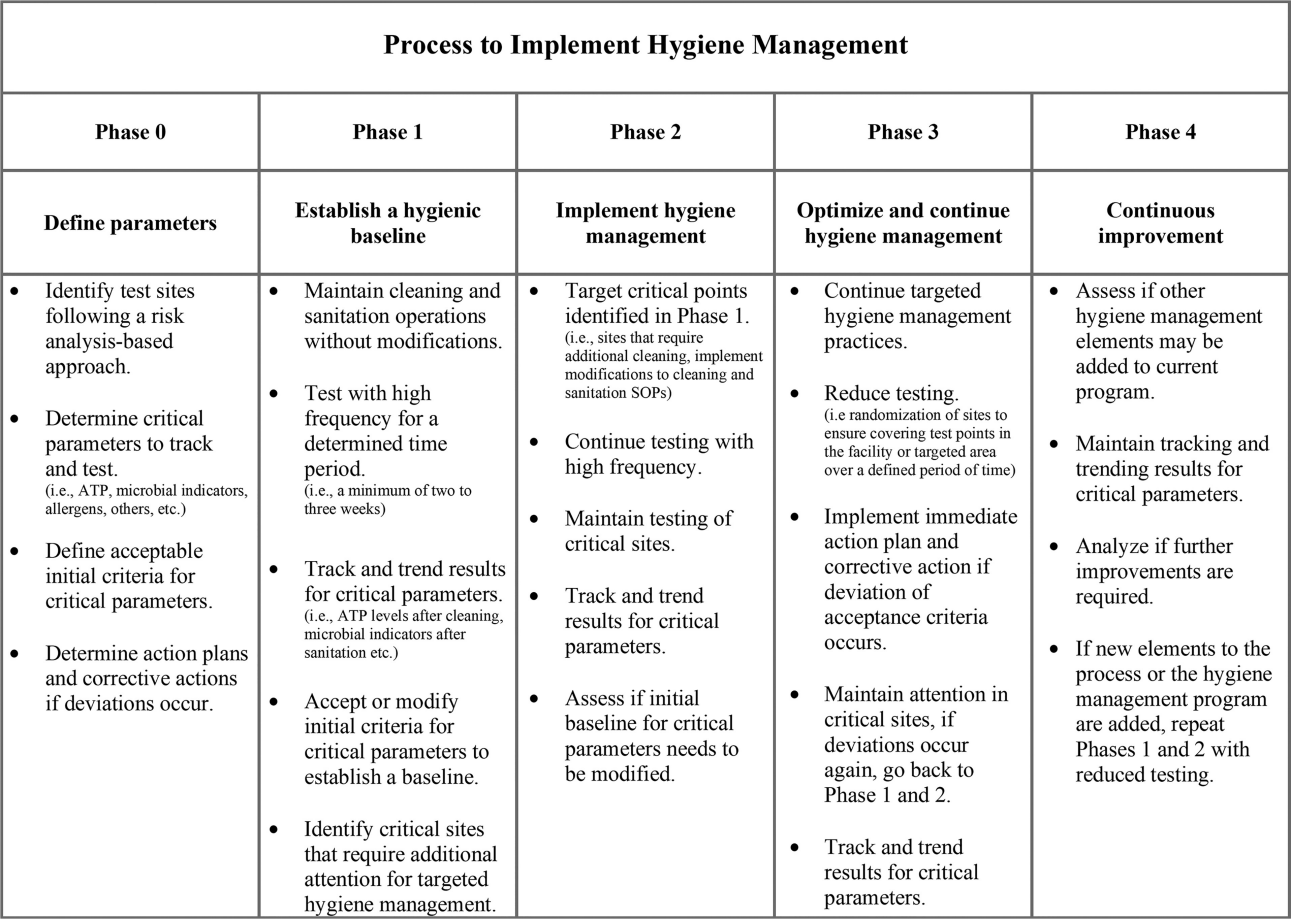
Conceptual overview of process to implement hygiene management.

Even though ATP testing is usually performed after every cleaning and sanitation operation, microbial quantification should be performed periodically in addition to ATP testing to verify that sanitation is effective. The use of both microbial and ATP monitoring can provide a robust set of data that verifies the efficacy of SSOPs (9). To effectively verify and improve cleaning and sanitation processes, hygiene monitoring programs need to address the frequency of testing, the location of test sites, and actionable limits for tests. These considerations are product and process specific but require a systematic framework to implement. Selecting sampling sites may require mapping the complete facility and production process, dividing the facility into zones based on microbiological risk to the product, and completing an assessment of the most appropriate test sites (8). Test sites should be selected after conducting an appropriate risk analysis to understand the risks associated with sites given the processing stage, proximity to food, potential for cross-contamination, ease of cleaning, and condition of the surface being tested (17). A conceptual overview of the process to implement hygiene monitoring is presented in Fig. 1.

In this study, targeted cleaning directed by site-specific ATP bioluminescence detection was implemented as a measure to improve environmental cleanliness of a tofu production facility. This process was chosen for study because tofu production applies few hurdles to control for microbial growth and is therefore particularly sensitive to spoilage (18). Thus, it was hypothesized that targeted improvements to the cleanliness of the processing environment, monitored by ATP luminescence detection, would improve the microbiological quality of products and the processing environment. This study was conducted over three phases (Fig. 1) as follows: establishment of a baseline hygiene level of the plant and products without targeted cleaning (phase 1), implementation of targeted cleaning practices directed by ATP results from phase 1 while maintaining extensive ATP testing to verify efficacy (phase 2), and maintenance of cleaning and sanitation practices with reduced ATP testing (phase 3). ATP testing was complemented by culture-based testing of environmental and product samples for the following three groups of target microorganisms: total aerobic microorganisms, yeasts and mold, and lactic acid bacteria. These groups were selected because they are common measures of environmental cleanliness and because they often cause food spoilage (19–21).

## RESULTS

### Environmental quality.

ATP, yeasts and molds, lactic acid bacteria, and aerobic microorganisms were quantified from swabs at 30 predetermined sites (21 zone 1 sites and 9 zone 2 sites) and then transformed to binary pass/fail results based on predetermined cutoffs specific to each measure to determine the impact of targeted cleaning on the hygiene of the processing environment. After excluding data due to excessive sample processing time (>6 days), a total of 5,196 measurements were retained across all phases of the study.

**FIG 2:**
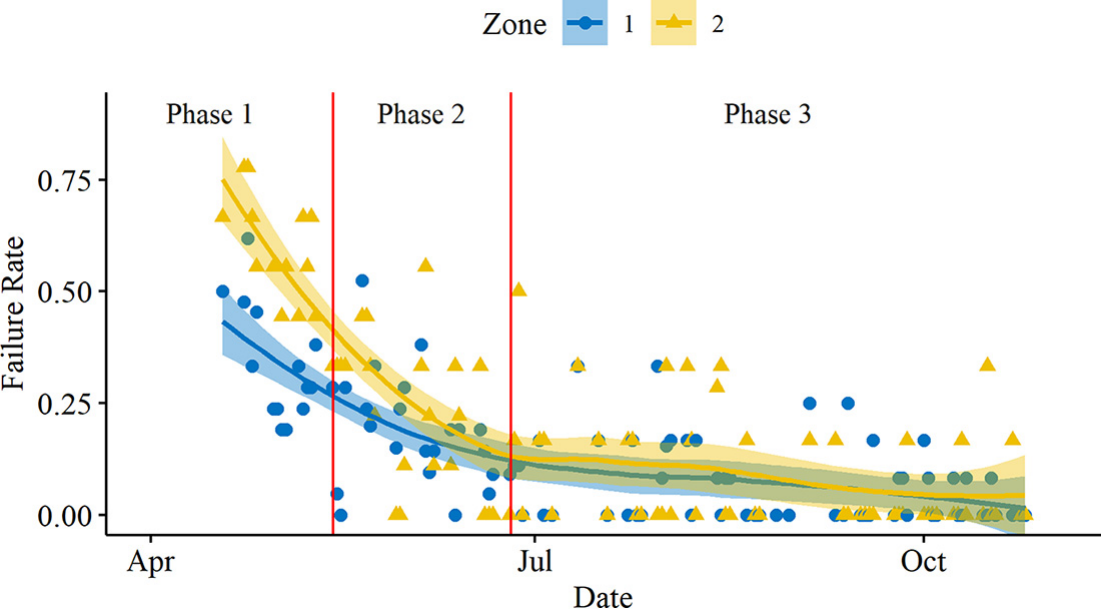
ATP swab failure rate over time. Trend lines are locally fitted polynomial regressions computed via the LOESS method, grouped by zone. Vertical lines correspond to the separation of the three phases utilized in this study (phase 1, preintervention—30 sites targeted per day [21 zone 1 and 9 zone 2]; phase 2, postintervention—30 sites targeted per day [21 zone 1 and 9 zone 2]; and phase 3, postintervention—18 randomized sites targeted per day [12 zone 1 and 6 zone 2]).

**FIG 3:**
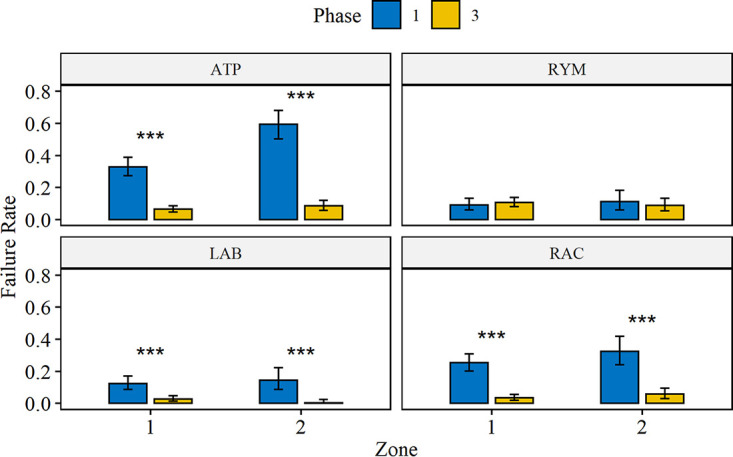
Proportion of swabs, aggregated by zone, failing to meet the minimum sanitary requirements based on the measurement of ATP, yeasts and molds (RYM), lactic acid bacteria (LAB), and aerobic microorganisms (RAC) during phases 1 and 3. Error bars represent the 95% confidence interval for each group based on the binomial distribution. Asterisks correspond to a significant difference between phases for a given zone (*P* < 0.001, Fisher’s exact test).

Over the course of the study, the proportion of sites failing to meet the minimum sanitary requirement day to day, based on ATP swabs, was highest during phase 1 and then steadily decreased during phase 2 before leveling off in phase 3 (Fig. 2); this indicated that targeted cleaning was improving the cleanliness of the facility. When aggregated by zone, the results show that targeted cleaning significantly lowered the proportion of swabs that failed to meet the minimum sanitary requirements between phases 1 and 3 for lactic acid bacteria and aerobic microorganisms in both zones 1 and 2 but did not significantly change the proportion of swabs that failed to meet the minimum sanitary requirement for yeasts and molds in either zone 1 or 2 (*P* < 0.05, Fisher’s exact test) (Fig. 3). The reduction between phases 1 and 3 was larger for aerobic microorganisms (21.8% for zone 1, 26.8% for zone 2) compared to that of lactic acid bacteria (9.7% for zone 1, 14.1% for zone 2) and was reflected in the significantly lower proportion of ATP swabs that failed to meet the minimum sanitary requirements in both zones 1 and 2 (*P* < 0.05, Fisher’s exact test) (Fig. 3). The reduction between phases 1 and 3 was largest for ATP swabs among all of the tests (26.5% for zone 1, 51.0% for zone 2). Finally, there was a significantly higher proportion of swabs in zone 2 that failed to meet the minimum sanitary requirements compared to zone 1 for only two groups: ATP swabs in phase 1 and lactic acid bacteria swabs in phase 3 (though the difference between zones 1 and 2 for lactic acid bacteria was small—2.4%).

When aggregated by site, as opposed to zone, targeted cleaning caused a decrease in the proportion of swabs failing to meet the minimum sanitary requirements between phases 1 and 3 for the majority of sites (Table 1). Though some sites exhibited an increase in the proportion of failing swabs, these increases were not significant (*P* > 0.05, Fisher’s exact test). There was a significant decrease in the proportion of swabs that failed to meet the minimum sanitary requirement for two sites when quantifying lactic acid bacteria (1 in zone 1, 1 in zone 2) and nine sites when quantifying aerobic microorganisms (6 in zone 1, 3 in zone 2); targeted cleaning did not result in a significant decrease in the proportion of swabs that failed to meet the minimum sanitary requirement for any sites when measuring yeasts and molds (*P* < 0.05, Fisher’s exact test). Similar to when data were aggregated by zone, the significant decrease in the proportion of swabs failing to meet the minimum sanitary requirements across specific sites for lactic acid bacteria and aerobic microorganisms was reflected in 14 sites that showed a significant decrease in the proportion of swabs failing to meet the minimum sanitary requirement when measuring ATP (9 in zone 1, 5 in zone 2). The reduction in the proportion of failing swabs for all metrics between the pre- and postintervention phases of study across all sites is presented in Table 1.

**TABLE 1:** Reduction in the proportion of swabs failing to meet the minimum sanitary requirements across all sites and measurements between phases 1 and 3

Zone	Site no. and description	ATP		RYM		LAB		RAC	
		Reduction (%)^*a*^	Significance^*b*^	Reduction (%)	Significance	Reduction (%)	Significance	Reduction (%)	Significance
1	01. Soybean hopper corner	49.6	***	24.9	ns	79.1	***	−15.8	ns
	02. Auger shaft-flexicon	52.9	***	−7.9	ns	−5.5	ns	12.6	ns
	03. Slurry tank inside	0.0	ns	0.0	ns	7.7	ns	15.4	ns
	04. Bulk (soymilk) tank inside	23.1	ns	0.0	ns	7.7	ns	23.1	ns
	05. Bulk (soymilk) tank agitator	92.3	***	−7.9	ns	15.4	ns	20.4	*
	06. Roller extractor shaft	30.8	*	3.1	ns	23.1	ns	24.1	ns
	07. Roller extractor roller	38.5	*	9.1	ns	9.1	ns	24.5	ns
	08. Bucket inside	40.9	*	1.8	ns	−5.9	ns	23.1	ns
	09. Bucket agitator	23.1	ns	−15.9	ns	0.0	ns	38.5	*
	10. Bucket turbulent stick	56.2	***	−2.6	ns	7.7	ns	38.5	***
	11. Curd holding tank/curd transfer barrel	47.9	***	−3.0	ns	5.1	ns	12.8	ns
	12. Conveyor belt white mat/auto press belt	20.3	ns	−0.2	ns	0.0	ns	15.4	ns
	13. Conveyor green plastic side belt	7.7	ns	1.8	ns	23.1	ns	30.8	*
	14. Chain conveyor/transfer conveyor	49.1	**	1.8	ns	0.0	ns	15.4	ns
	15. Chilling tank smooth surface/conveyor tank	0.0	ns	−11.8	ns	0.0	ns	7.7	ns
	16. Chilling tank inside corner	0.0	ns	0.0	ns	0.0	ns	0.0	ns
	17. Chilling tank conveyor	23.1	ns	−12.3	ns	0.0	ns	15.4	ns
	18. Chilling tank roller shaft	0.0	ns	−7.7	ns	23.1	ns	38.5	*
	19. Chilling tank roller sprocket	16.7	ns	16.8	ns	23.1	ns	38.5	*
	20. Overflow tofu tank inside/ rolling tanks	25.8	ns	−3.4	ns	7.7	ns	−3.4	ns
	21. Overflow tofu tank corner	7.7	ns	−7.1	ns	0.0	ns	0.0	ns
2	50. MV4 HMI screen	85.6	***	10.1	ns	7.7	ns	4.9	ns
	51. MV4 HMI screen control button and E-stop	−5.5	ns	10.0	ns	15.4	ns	10.0	ns
	52. MV4 film rollers	89.2	***	−0.6	ns	7.7	ns	20.3	ns
	53. Rolling rack	40.5	*	3.1	ns	7.7	ns	23.1	ns
	54. Rolling rack trays	−11.8	ns	2.1	ns	7.7	ns	10.8	ns
	55. MV side rail	23.8	ns	1.4	ns	23.1	ns	26.0	ns
	56. Chiller tank outside/conveyor tank outside	9.7	ns	−11.1	ns	7.7	ns	30.8	*
	57. Waterpack control panel buttons	76.5	***	−10.8	ns	28.1	*	51.1	***
	58. Waterpack upper guide rails prior to sealer	38.5	**	8.7	ns	23.1	ns	61.5	***

a Reduction calculated as *P*_fail, P1_ − *P*_fail, P3_.

b Significance according to Fisher’s exact test; ns, not significant; *, *P* < 0.05; **, *P* < 0.01; ***, *P* < 0.001.

### Microbiological product quality.

**FIG 4:**
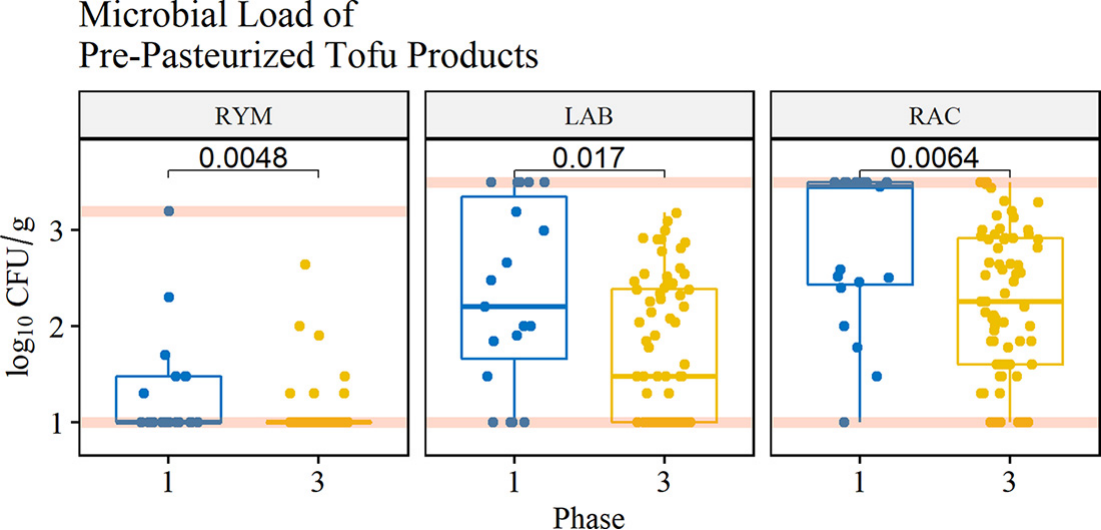
Microbial load of yeasts and mold (RYM), lactic acid bacteria (LAB), and aerobic microorganisms (RAC) in packaged tofu products sampled prepasteurization. Red lines correspond to the limits of detection for each group of interest. Numbers correspond to the *P* value between phases 1 and 3.

**FIG 5:**
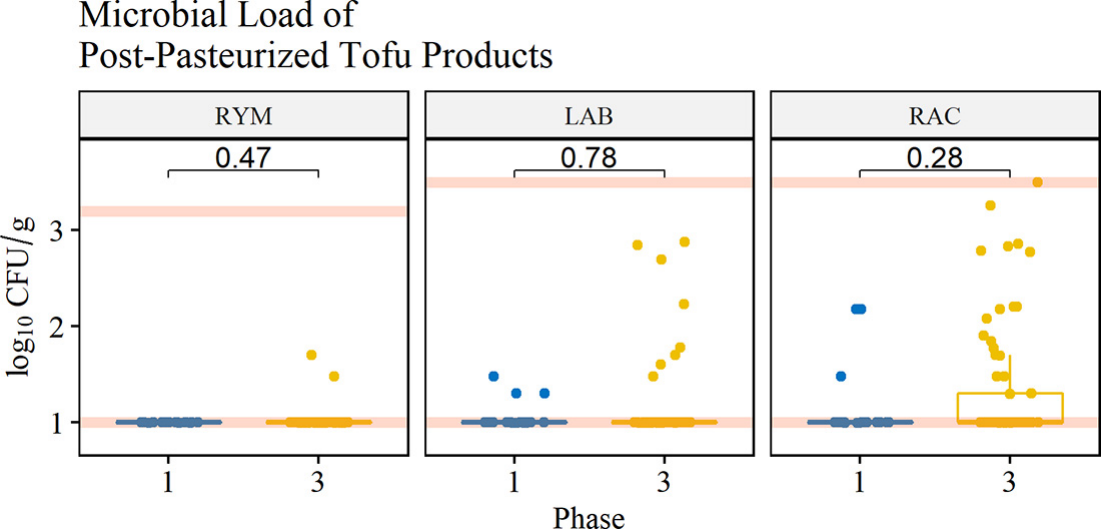
Microbial load of yeasts and mold (RYM), lactic acid bacteria (LAB), and aerobic microorganisms (RAC) in packaged tofu products sampled postpasteurization. Red lines correspond to the limits of detection for each group of interest. Numbers correspond to the *P* value between phases 1 and 3.

Tofu products taken from the line during production were sampled for yeasts and molds, lactic acid bacteria, and aerobic microorganisms to determine the impact of targeted cleaning on the quality of finished products. Among prepasteurized products, the mean rank log_10_ CFU per gram of postintervention products (*n* = 68) was significantly lower than that of preintervention products (*n* = 19) for yeasts and mold, lactic acid bacteria, and aerobic microorganisms (*P* < 0.05, Mann-Whitney U test) (Fig. 4). Among postpasteurized products, the mean rank log_10_ CFU per gram of postintervention products (*n* = 69) was not significantly different than preintervention products (*n* = 19) for yeasts and mold, lactic acid bacteria, and aerobic microorganisms (*P* > 0.05, Mann-Whitney U test) (Fig. 5).

### ATP and microbiological swab agreement.

Because microbiological swabs were taken immediately adjacent to ATP swabs, it was possible to analyze *post hoc* the agreement between ATP swabs and microbiological swabs at those sites. In total, 960 samples over the pre- and postintervention phases of study were quantified for both ATP and viable microorganisms. Data were transformed to binary pass/fail results based on the same predetermined cutoffs as previously mentioned, but rather than analyze each microbiological group individually (i.e., yeasts and molds, lactic acid bacteria, and aerobic microorganisms), a binary transformation was made such that if any of the microbiological measurements exceeded their respective cutoffs, then the site failed to meet the minimum sanitary requirement. For a site to pass the minimum microbiological sanitary requirements, every microbiological group had to be below its individual predetermined cutoff. ATP and microbiological swab results agreed for 75.1% (72.3 to 77.7%) of samples (95% confidence interval [CI], sampling distribution). ATP swab results failed the minimum sanitary requirements but microbiological swabs passed for 11.7% (9.8 to 13.9%) of samples (95% CI, sampling distribution), and ATP swabs passed the minimum sanitary requirements but microbiological swabs failed for 13.2% (11.2 to 15.5%) of samples (data not shown).

## DISCUSSION

Cleaning and sanitation procedures are critical in the food industry and are the first line of defense to prevent contamination of food products from the production environment. They are required to prevent the presence and proliferation of pathogenic and spoilage microorganisms. In this study, cleaning and sanitation operations were monitored in a soy-based manufacturing production facility over a period of 3 weeks utilizing both ATP bioluminescence and microbial indicators to assess the cleaning efficacy. After this time, targeted cleaning of specific sites that showed the highest rate of cleaning and sanitation failures was implemented; the effect of targeted cleaning was monitored with the same verification methods.

### Environmental quality.

The results from this study indicate that targeted cleaning directed by ATP monitoring may improve the environmental hygiene of food-processing facilities (Fig. 2); this was verified by microbiological tests. A significant decrease in the proportion of swabs failing to meet the minimum sanitary requirements for lactic acid bacteria and aerobic microorganisms indicates that the targeted cleaning applied after phase 1 had a positive effect on the facility’s hygiene. In contrast, the hygiene measure for yeasts and molds remained unchanged with targeted cleaning efforts. However, it is important to consider that equipment surfaces may not be the only sources of yeast and molds. Other sources of yeasts and molds include air, raw materials, and packaging, which would likely be unaffected by improvements to cleaning and sanitation (19).

There was a minimal difference between the microbial indicator failure rates between zones 1 and 2 over the course of the study. However, there was a significant difference between failure rates of zone 1 and zone 2 sites for ATP during phase 1 that was absent in phase 3. Though the data do not suggest that zone 2 surfaces were less hygienic than zone 1 with regard to microorganisms, the insignificant difference for ATP between zone 1 and zone 2 sites during phase 3 suggests that the plant was overall cleaner. In this case, cleaner zone 2 surfaces corresponded with improved plant hygiene, which was reflected in the improvement of hygiene measures associated with aerobic microorganisms and lactic acid bacteria. Such an effect protects against the establishment of spoilage and likely pathogenic microorganism populations (22).

This study also showed that ATP monitoring and the use of microbiological indicators may result in more effective equipment surface cleaning. When comparing individual sites (Table 1), targeted cleaning was most effective in specific portions of the manufacturing line. A total of 14 sites for ATP and 9 sites for aerobic microorganisms showed a significant reduction in the proportion of failing swabs between phases 1 and 3. Some sites exhibited an increase in the proportion of failing swabs, but these increases were not significant. Taken together, these data indicate that targeted cleaning may only improve hygiene for specific sites and that other factors aside from cleaning (e.g., equipment geometry, temperature of product at the processing step, etc.) may have a greater effect on the hygienic quality of other sites.

For this plant, ATP monitoring and the use of total aerobic count best reflected (greatest change) the effect of targeted cleaning; these could be selected as methods for routine verification of cleaning and sanitation operations in this facility. These two measures may not apply to all products and facilities. It is important for facilities to choose metrics that are sensitive to changes in the plant environment and can easily detect deviations to cleaning and sanitation. Setting critical parameters for monitoring ATP as well as microbiological criteria on equipment surfaces is highly dependent on the manufacturing site, design and state of the equipment, product, process, and cleaning process. Relying on data that can be trended over time is often helpful to establish an appropriate baseline. Baseline testing for ATP and microbiological parameters should be periodically reviewed and reassessed to verify that cleaning and sanitation operation procedures remain effective.

### Microbiological product quality.

In this study, there was an improvement in the microbial load of yeasts and molds, lactic acid bacteria, and aerobic microorganisms for prepasteurized products (Fig. 4) but not postpasteurized products after implementation of targeted cleaning (Fig. 5). It is important to note that for this process, the product undergoes two thermal processing steps. Soy milk requires an extraction that occurs at 88°C, which significantly reduces the microbial load associated with the raw materials; after this process, coagulated product is pressed, cut, and packaged before it is pasteurized in-package. Because the microbial load is reduced in soymilk during extraction, the microbial load of products determined in this study in prepasteurized products is primarily associated with postprocess contamination, including contact with equipment surfaces. The microbial load of postpasteurized products was not significantly different, which is expected as the packaged product is heat treated again, killing vegetative cells. The results from this study indicate that targeted cleaning monitored by ATP bioluminescence and microbial indicators could improve microbiological product quality for products that do not undergo an in-package pasteurization step (e.g., fresh fruits and vegetables).

### Hygiene monitoring with ATP bioluminescence.

ATP bioluminescence was utilized as a tool for hygiene monitoring during this study. Prior to implementation of targeted cleaning, site selection was done to identify areas in the production environment that were most likely to pose challenges during cleaning and sanitation. Site selection should comprehensively cover the production environment such that results obtained during monitoring activities reflect the cleanliness of the area (23). ATP bioluminescence monitoring accompanied by microbial evaluation demonstrated the effectiveness of targeted cleaning. Significant differences were determined between the proportion of swabs failing to meet the minimum sanitary requirements set for both ATP and microbiological parameters for specific sites (Table 1). In cases where ATP did not agree with the microbiological result, it is important to consider sampling area (which although adjacent could display differences) as well as the microbial load required for ATP detection (approximately 10^3^ to 10^4^ CFU) (24).

Overall, the ATP data over time showed a decrease in the proportion of failing swabs as phases 2 and 3 were implemented (Fig. 2). Evaluation of ATP swabs as a tool to identify microbial contamination showed that 75.1% (95% CI, 72.3 to 77.7%) of ATP swabs reflected the microbiological status of test sites. This correlation is likely due to inadequate sanitation resulting from poor cleaning or adequate sanitation resulting from proper cleaning. Although ATP generally does not directly correlate to the number of microorganisms on a given surface (16, 25), it can be used as a rapid tool to assess equipment cleanliness (26). ATP sources are not exclusive to microbial ATP; food residues, which cannot be detected by microbiological tests, can also account for failing ATP swabs. In this study, this occurred when the microbial counts passed the minimum sanitary requirements, but the ATP levels did not—11.7% (9.8 to 13.9%). The ATP from nonbacterial sources can evidence potential niches where food residues may accumulate over time and thus enable microbial growth. The need to conduct both biochemical and microbiological tests is especially highlighted where the microbiological results did not meet the minimum sanitary requirements but ATP did—13.2% (11.2 to 15.5%). In these cases, spores may cause the discrepancy because they do not produce ATP while in spore form (27, 28) but can still enter packages and induce spoilage after germination.

ATP is considered a method to rapidly verify cleaning while microbial tests provide results to verify the sanitation status; both tests can provide a more holistic assessment of the effectiveness of cleaning and sanitation operations. ATP swabs alone can provide rapid and robust daily verification for cleaning and sanitation operations of a facility. In this study, ATP swabs taken after cleaning and sanitation would have either correctly verified the microbiological hygiene status or elicited additional cleaning due to food residues in 86.8% (84.5 to 88.8%) of cases. Microbiological testing could supplement this on a less frequent basis (e.g., weekly) to ensure continued efficacy of cleaning and sanitation.

### Conclusion.

This study showed that the microbiological quality of products improved following targeted cleaning implemented to improve the hygiene of the production environment. ATP bioluminescence and microbial indicators seem to be effective tools to monitor cleaning and sanitation operations and to direct the efforts of the cleaning and sanitation crew. Further, these results indicate that both biochemical and microbiological tests should be used to monitor hygiene, as they are complementary in efficiently assessing the cleaning and sanitary status of the manufacturing environment and processed products. This study serves as a framework for companies to implement hygiene monitoring in their own facilities, but it is important to note that different products and plants may require different tests and/or critical limits to the tests used in this study.

## MATERIALS AND METHODS

### Facility and process overview.

This study was conducted in a medium-sized facility that produces only soy-based products. In brief, the tofu produced at this facility is made from coagulated soymilk extracted from hydrated soybeans; extraction occurs at approximately 88°C (190°F). After coagulation, the curd is pressed, cut, water-cooled, and vacuum packaged for retail or institutional use (prepasteurization). After packaging, products are pasteurized in package, water-cooled, and refrigerated for cold chain distribution (postpasteurization); the shelf life of products is declared as 60 days. The facility in which this study was conducted was chosen due to a professional relationship between the authors and facility management.

### Study design and implementation.

The goal of this study was to investigate the impact of targeted cleaning on the microbial quality of the environment and finished products. Microbiological environmental and product testing occurred in two phases for analysis: preintervention (phase 1) and postintervention (phase 3) of targeted cleaning activities. Prior to the start of study, 30 sites (21 zone 1 sites and 9 zone 2 sites) were identified by the authors and facility management for ATP monitoring and microbiological enumeration. These sites were chosen based on phase of production and relative cleaning and sanitation difficulty (i.e., sites deemed harder to clean were favored for testing over others); the sites and zone designations are listed in Table 1. ATP monitoring occurred over three phases; phase 1 (baseline assessment), verification of cleaning and sanitation procedures utilizing extensive ATP testing (30 sites targeted per day); phase 2, postimplementation of targeted cleaning, maintaining extensive ATP testing (30 sites targeted per day); and phase 3, postimplementation with maintenance of cleaning and sanitation practices and reduced ATP testing (18 randomized sites targeted per day). Reduction and randomization of sites for ATP testing conducted in phase 3 were performed utilizing the 3M Clean-Trace data management software (v 1.3.0.0) with the randomization function.

To establish a baseline hygiene level of the facility and products, the preintervention phase of the study occurred over 3 weeks following the facility’s normal cleaning and sanitation program. After this, an adjustment period of 6 weeks was allowed for the cleaning and sanitation crew to adopt targeted cleaning practices indicated by phase 1 data. Targeted cleaning practices incorporated results from ATP and microbiological testing, described below, into the cleaning and sanitation program. Management informed the crew of which sites consistently had high levels (RLU/swab > 500) of ATP during the preintervention phase, and the cleaning and sanitation crew subsequently targeted those sites for enhanced cleaning. Enhanced cleaning included increased time spent cleaning portions of the line associated with the preidentified sites and some disassembly of equipment to access hard-to-clean areas. Sanitation proceeded as usual after cleaning. Following the adjustment period, the postintervention phase of study occurred over 16 weeks to determine the impact of targeted cleaning.

### Biochemical and microbiological testing.

During phases 1, 2, and 3, ATP was quantified at the 30 predetermined sites using 3M Clean-Trace surface ATP swabs (3M Company, St. Paul, MN) and the 3M Clean-Trace hygiene monitoring system LM1 luminometer (v 1.1.0.0) (3M Company, St. Paul, MN) immediately following cleaning and sanitation according to the manufacturer’s instructions. During phases 1 and 3, yeasts and molds, lactic acid bacteria, and aerobic microorganisms were quantified from a single complimentary 3M Quick Swab (3M Company, St. Paul, MN) taken adjacent (<15 cm or 6 in.) to the area swabbed for ATP. Samples were collected by swabbing in two directions, horizontally and vertically, an area of approximately 100 cm^2^. Both the ATP and microbiological swabs were taken by a trained member of the cleaning and sanitation crew; thus, the swabbing portion of this study represents “real-world” execution. Microbiological swabs were kept refrigerated (<4°C) and processed within 1 to 6 days (dictated by transportation time, shift, and day of the week). Swabs were serially diluted in Butterfield’s buffer (3M, St. Paul, MN) and plated onto 3M Petrifilm plates according to the manufacturer’s instructions as follows: rapid yeast and mold count plates (RYM; 3 days at 25°C), lactic acid bacteria count plates (LAB; 2 days at 30°C), and rapid aerobic count plates (RAC; 1 day at 35°C).

Two packaged products from each production lot, one prepasteurized and the other postpasteurized, were taken directly from the production line during phases 1 and 3. These products were kept refrigerated (<4°C) and microbiologically characterized within 1 to 6 days. In brief, one 25-g subsample of tofu was aseptically removed from each packaged product and stomached in a sterile filter bag with 225 ml 0.1% peptone water (Becton, Dickinson, Franklin Lakes, NJ); stomaching occurred at 200 rpm for 90 s. Samples were serially diluted with Butterfield’s buffer and plated onto RYM, LAB, and RAC plates according to the manufacturer’s instructions (see above).

### Data and statistical analyses.

Quantitative data collected from ATP and microbiological swabs were transformed to binary pass/fail values with the following cutoffs, i.e., the minimum sanitary requirements (specific to this study), as follows: ATP − RLU/swab (100 cm^2^) > 500, RYM − log CFU/swab > 1.30 (20 CFU), LAB − log CFU/swab > 2.30 (200 CFU), and RAC − log CFU/swab > 2.30. Measurements that exceeded these cutoffs failed the test, indicating that the site was not adequately cleaned and sanitized for processing, i.e., these swabs failed to meet the minimum sanitary requirements. These values were chosen based on manufacturer recommendations, in coordination with plant management, and based on the authors’ experience. They are in alignment with previously established levels (26, 29), but it is important to note that present day hygiene monitoring emphasizes risk-based decision making and thus these cutoffs will vary depending on the product and process (30). Quantitative data obtained from microbiological analysis of finished products were not transformed except that for values less than or greater than the limit of detection (based on the chosen dilutions and countable ranges for each of the utilized media) were set to the limit of detection.

Data analysis was conducted in R (v 4.0.2) (31) using R Studio (v 1.3.1073) (32) with the following packages: readxl (v1.3.1) (33), dplyr (v 1.0.2) (34), ggpubr (v 0.4.0) (35), tidyr (v 1.1.1) (36), and kableEztra (v 1.2.1) (37). Trending data were visualized via locally estimated scatterplot smoothing (LOESS) with the following parameters: span = 0.75, degree = 2, and confidence interval = 95%. All statistical tests were conducted as two-sided tests with an α value of 0.05. Binary data were analyzed using the binomial distribution to obtain 95% confidence intervals for groups; groups were compared using Fisher’s exact test. Quantitative product data were compared using the nonparametric Mann-Whitney U test due to the skewed nature of the data. Agreement between the results from ATP swabs and microbiological swabs was analyzed using the sampling distribution to obtain 95% confidence intervals for groups.

### Data availability.

The data and code used to draw conclusions in this study are deposited in a Zenodo repository at https://doi.org/10.5281/zenodo.4287499 (38).
